# Supplementary material for: Antiretroviral therapies and status of people living with HIV in Japan: An update from hospital survey and national database
Source: PLoS One. 2025 Jan 27;20(1):e0317655. doi: 10.1371/journal.pone.0317655 (PMC11771938; doi:10.1371/journal.pone.0317655)
Supplement: S1 Table — (DOCX) [file pone.0317655.s001.docx]

**S1 Table.** Antiretroviral therapy codes

| **Drug code** | **Trade name** | **Generic name** | **Abbreviation_1** | **Abbreviation_2** |
| --- | --- | --- | --- | --- |
| 620005140 | Retrovir Capsule 100 mg | Zidovudine | AZT(ZDV) | AZT(ZDV) |
| 610443081 | Videx EC Capsule 125 mg | Didanosine | ddL | ddL |
| 610443082 | Videx EC Capsule 200 mg | Didanosine | ddL | ddL |
| 620000425 | Epivir Tablet 150 mg | Lamivudine | 3TC | 3TC_150 |
| 620000426 | Epivir Tablet 300 mg | Lamivudine | 3TC | 3TC_300 |
| 620004975 | Crixivan Capsule 200 mg | Indinavir sulfate | IDV | IDV |
| 610412192 | Zerit Capsule 15 mg | Saquinavir | d4T | d4T |
| 610412193 | Zerit Capsule 20 mg | Saquinavir | d4T | d4T |
| 620003516 | Invirase Capsule 200 mg | Mesylate of saquinavir | SQV | SQV |
| 620004347 | Invirase Tablet 500 mg | Mesylate of saquinavir | SQV | SQV |
| 621143601 | Norvir Oral Solution 8% 80 mg | Ritonavir | RTV | RTV |
| 622054801 | Norvir Tablet 100 mg | Ritonavir | RTV | RTV |
| 621143701 | Viracept Tablet 250 mg | Mesylate of nelvirapine | NFV | NFV |
| 610421341 | Viramune Tablet 200 mg | Nevirapine | NVP | NVP |
| 620004998 | Ziagen Tablet 300 mg | Abacavir | ABC | ABC_300 |
| 620006943 | Stocrin Tablet 600 mg | Efavirenz | EFV | EFV |
| 621932401 | Stocrin Tablet 200 mg | Efavirenz | EFV | EFV |
| 620000454 | Reyataz Capsule 150 mg | Atazanavir sulfate | ATV | ATV |
| 620000455 | Reyataz Capsule 200 mg | Atazanavir sulfate | ATV | ATV |
| 620001903 | Viracept Tablet 300 mg | Tenofovir disoproxil fumarate | TDF | TDF |
| 620002465 | Lexiva Tablet 700 mg | Fosamprenavir calcium hydrate | FPV | FPV |
| 620002488 | Emtriva Capsule 200 mg | Emtricitabine | FTC | FTC |
| 620005884 | Prezista Tablet 300 mg | Darunavir | DRV_300 | DRV_300 |
| 621930301 | Prezista Naive Tablet 400 mg | Darunavir | DRVn_400 | DRV_400 |
| 622276701 | Prezista Naive Tablet 800 mg | Darunavir | DRVN_800 | DRV_800 |
| 622403501 | Prezista Tablet 600 mg | Darunavir | DRV_600 | DRV_600 |
| 620007815 | Isentress Tablet 400 mg | Raltegravir | RAL_400 | RAL_400 |
| 622626001 | Isentress Tablet 600 mg | Raltegravir | RAL_600 | RAL_600 |
| 620009086 | Intelence Tablet 100 mg | Etravirine | ETR | ETR |
| 620009087 | Celsentri Tablet 150 mg | Maraviroc | MVC | MVC |
| 622149101 | Edurant Tablet 25 mg | Rilpivirine | RPV | RPV |
| 622336201 | Tivicay Tablet 50 mg | Dolutegravir sodium | DTG | DTG |
| 622702801 | Pifeltro Tablet 100 mg | Doravirine | DOR | DOR |
| 621144201 | CombiVir Combination Tablet | Zidovudine | COM | AZT(ZDV) |
|  |  | Lamivudine |  | 3TC |
| 621384201 | Kaletra Combination Oral Liquid | Lopinavir | LPV/r | LPV |
|  |  | Ritonavir |  | RTV |
| 621765701 | Kaletra Combination Tablet | Lopinavir | LPV/r | AZT(ZDV) |
|  |  | Ritonavir |  | RTV |
| 621657001 | Epzicom Combination Tablet | Abacavir sulfate | EZC | ABC_600 |
|  |  | Lamivudine |  | 3TC_300 |
| 622699101 | Lamivudine Combination Tablet "Amer" | Abacavir sulfate | LBM | ABC_600 |
|  |  | Lamivudine |  | 3TC_300 |
| 621662301 | Truvada Combination Tablet | Tenofovir disoproxil fumarate | TVD | TDF |
|  |  | Emtricitabine |  | FTC |
| 622235801 | Stribild Combination Tablet | Elvitegravir | STB | EVG |
|  |  | Cobicistat |  | COBI |
|  |  | Emtricitabine |  | FTC |
|  |  | Tenofovir disoproxil fumarate |  | TDF |
| 622388001 | Complera Combination Tablet | Emtricitabine | CMP | FTC |
|  |  | Rilpivirine |  | RPV |
|  |  | Tenofovir disoproxil fumarate |  | TDF |
| 622408801 | Triumeq Combination Tablet | Dolutegravir sodium | TRI | DTG |
|  |  | Abacavir |  | ABC_600 |
|  |  | Lamivudine |  | 3TC |
| 622507701 | Genvoya Combination Tablet | Elvitegravir | GEN | EVG |
|  |  | Cobicistat |  | COBI |
|  |  | Emtricitabine |  | FTC |
|  |  | Tenofovir disoproxil fumarate |  | TDF |
| 622532301 | Prezcobix Combination Tablet | Darunavir | PCX | DRV_800 |
|  |  | Cobicistat |  | COBI |
| 622522501 | Descovy Combination Tablet HT | Tenofovir alafenamide fumarate | DVY-HT | TAF |
|  |  | Emtricitabine |  | FTC |
| 622522601 | Descovy Combination Tablet LT | Tenofovir alafenamide fumarate | DVY-LT | TAF |
|  |  | Emtricitabine |  | FTC |
| 622647301 | Odefsey Combination Tablet | Rilpivirine | ODF | RPV |
|  |  | Tenofovir alafenamide fumarate |  | TAF |
|  |  | Emtricitabine |  | FTC |
| 622660701 | Juluca Combination Tablet | Dolutegravir sodium | JUL | DTG |
|  |  | Rilpivirine |  | RPV |
| 622660501 | Bictarvy Combination Tablet | Bictegravir | BVY | BIC |
|  |  | Tenofovir alafenamide fumarate |  | TAF |
|  |  | Emtricitabine |  | FTC |
| 622683501 | Symtuza Combination Tablet | Darunavir | SMT | DRV_800 |
|  |  | Cobicistat |  | COBI |
|  |  | Emtricitabine |  | FTC |
|  |  | Tenofovir alafenamide fumarate |  | TAF |
| 622702901 | Dovato Combination Tablet | Dolutegravir sodium | DVT | DTG |
|  |  | Lamivudine |  | 3TC |

Abbreviation_1 is based on the trade names; Abbreviation_2 includes specifications on active ingredients’ contents. The numerical drug codes are established by the Japanese health authority.
