# Supplementary material for: Antiretroviral therapies and status of people living with HIV in Japan: An update from hospital survey and national database
Source: PLoS One. 2025 Jan 27;20(1):e0317655. doi: 10.1371/journal.pone.0317655 (PMC11771938; doi:10.1371/journal.pone.0317655)
Supplement: S2 Table — (a) Time course of parameters related to PLHIV status in Japan by ratio, 2016–2020. (b) Parameters related to PLHIV status in Japan by ratio, 2020. (c) Parameters related to PLHIV status across the nine Japanese regions by ratio, 2020. (d) Parameters related to PLHIV status according to the mean volume of outpatients per facility in Japan by ratio, 2020. > = 100: Facilities with more than 100 retained patients, 10–99: Facilities with 10–99 retained patients, 1–9: Facilities with 1–9 retained patients. (DOCX) [file pone.0317655.s002.docx]

**S2 Table****.** The proportion of Figure 3.

a. Time course of parameters related to PLHIV status in Japan by ratio, 2016-2020.

|  | 2016 (%) | 2017 (%) | 2018 (%) | 2019 (%) | 2020 (%) |
| --- | --- | --- | --- | --- | --- |
| Diagnosed | 100 | 100 | 100 | 100 | 100 |
| Retained in care | 95.6 | 95.9 | 96.1 | 96.6 | 96.8 |
| On ART | 96.5 | 95.4 | 97.9 | 97.9 | 97.7 |
| Virally suppressed | 99.3 | 99.4 | 99.5 | 99.6 | 99.7 |

b. Parameters related to PLHIV status in Japan by ratio, 2020.

|  | Global (%) |
| --- | --- |
| Retained in care | 100 |
| On ART | 97.7 |
| Virally suppressed | 99.7 |

c. Parameters related to PLHIV status across the nine Japanese regions by ratio, 2020.

|  | Hokkaido (%) | Tohoku (%) | Koshinetsu (%) | Capital Area (%) | Tokai (%) | Hokuriku (%) | Kinki (%) | Chugoku-Shikoku (%) | Kyusyu (%) |
| --- | --- | --- | --- | --- | --- | --- | --- | --- | --- |
| Retained in care | 100 | 100 | 100 | 100 | 100 | 100 | 100 | 100 | 100 |
| On ART | 97.7 | 97.8 | 98.6 | 98.2 | 95.6 | 99.2 | 97.4 | 96.4 | 98.6 |
| Virally suppressed | 100 | 99.8 | 99.6 | 99.7 | 99.6 | 99.6 | 99.7 | 99.4 | 99.9 |

d. Parameters related to PLHIV status according to the mean volume of outpatients per facility in Japan by ratio, 2020. >= 100: Facilities with more than 100 retained patients, 10-99: Facilities with 10-99 retained patients, 1-9: Facilities with 1-9 retained patients

|  | >=100(%) | 10-99(%) | 1-9(%) |
| --- | --- | --- | --- |
| Retained in care | 100 | 100 | 100 |
| On ART | 97.6 | 98.2 | 95.4 |
| Virally suppressed | 99.7 | 99.8 | 99.7 |
