# Supplementary figures and images for: Antiretroviral therapies and status of people living with HIV in Japan: An update from hospital survey and national database
Source: PLoS One. 2025 Jan 27;20(1):e0317655. doi: 10.1371/journal.pone.0317655 (PMC11771938; doi:10.1371/journal.pone.0317655)

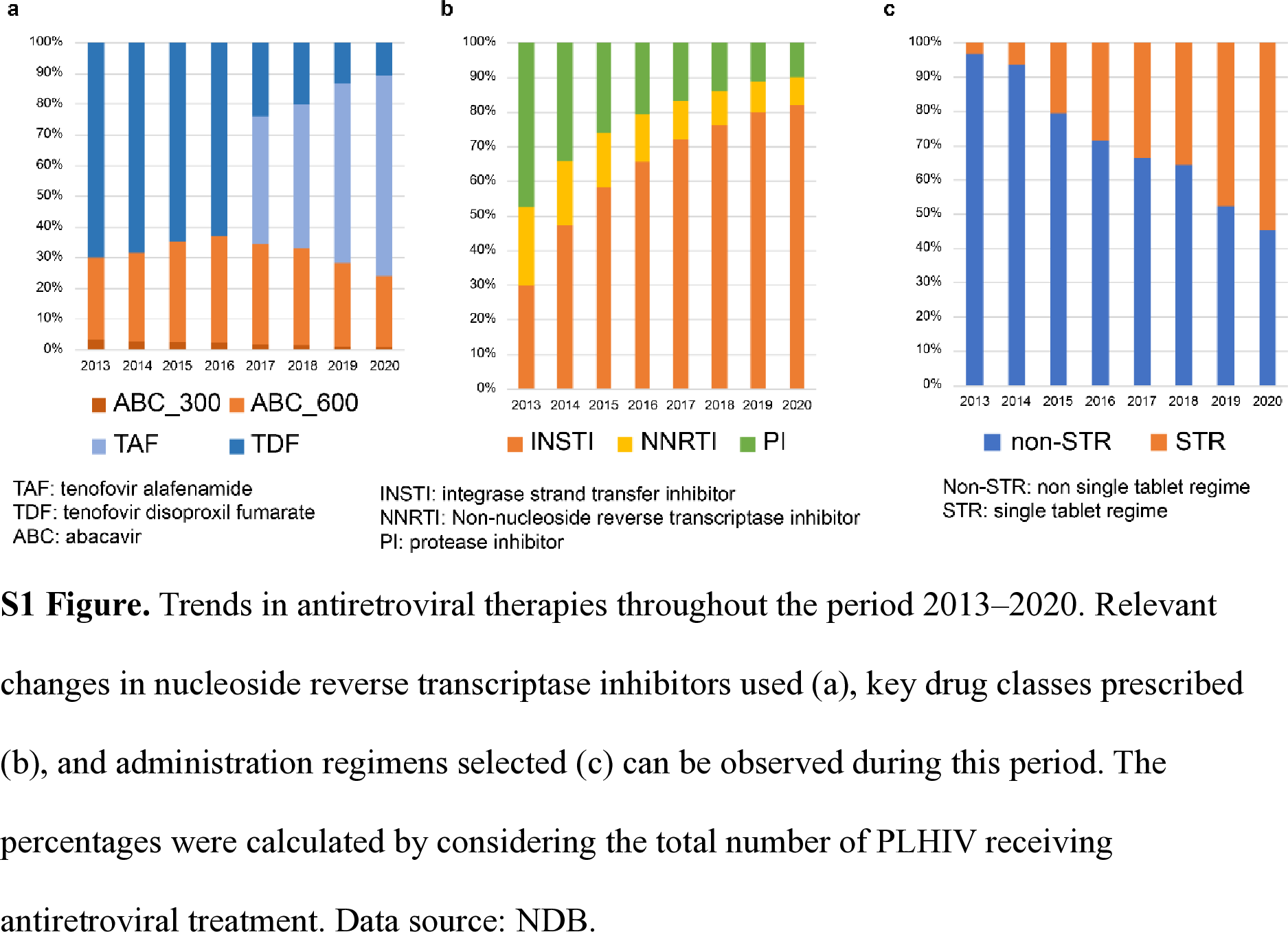

Supplement: S1 Fig — Relevant changes in nucleoside reverse transcriptase inhibitors used (a), key drug classes prescribed (b), and administration regimens selected (c) can be observed during this period. The percentages were calculated by considering the total number of PLHIV receiving antiretroviral treatment. Data source: NDB. (TIF) [file pone.0317655.s003.tif]

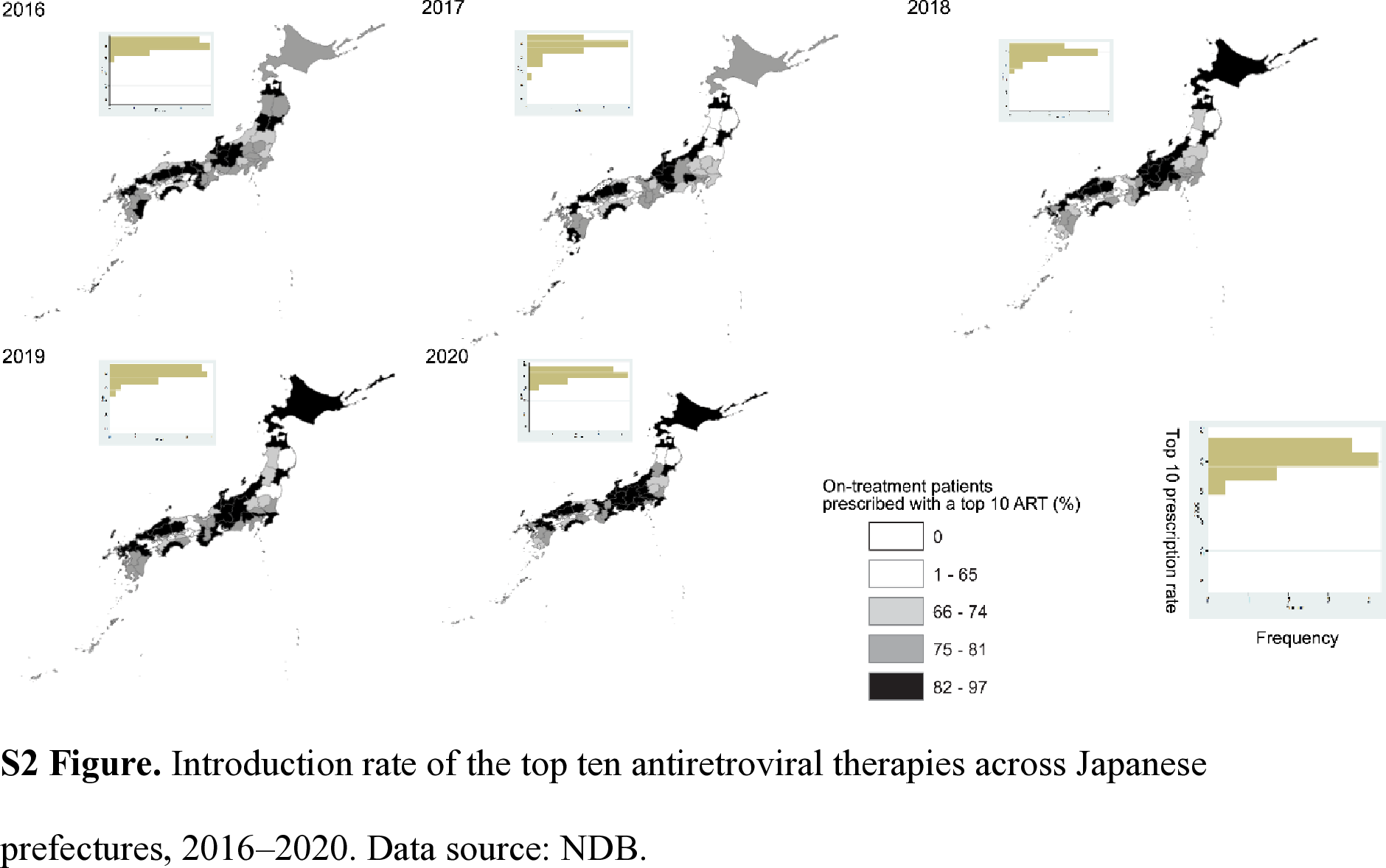

Supplement: S2 Fig — Data source: NDB. (TIF) [file pone.0317655.s004.tif]
