# Supplementary material for: Antiretroviral therapies and status of people living with HIV in Japan: An update from hospital survey and national database
Source: PLoS One. 2025 Jan 27;20(1):e0317655. doi: 10.1371/journal.pone.0317655 (PMC11771938; doi:10.1371/journal.pone.0317655)
Supplement: Attachment — Submitted filename: 20240715_v1.docx [file pone.0317655.s005.docx]

This study is about the overall epidemiology and ART use among PLHIV in Japan. It provided a variety of analytical results through a survey of a wide range of hospitals and access to the nationwide database. This is an interesting and necessary study, but I think it needs revision, especially including further clarification on some of the definitions.

Major comments

Abstract

AIDS patients -> PLHIV (also in the manuscript)

Introduction

I think it would be more appropriate to describe the detailed characteristics of NDB in the Methods section rather than in the Introduction section.

Methods

Line 101 Lists of the hospitals that participated in the survey and, if possible, of the respondents are required. It would be good to provide the information as a supplementary table.

Line 127 “Patient retained in care”

Please describe in detail what it means to be linked after diagnosis. For example, is this the same as the definition in the NDB of having visited a hospital or clinic at least once within a 6-month period? Also, I am curious about how cases are handled if PLHIV are transferred to another hospital while receiving treatment at a core hospital.

In addition, please provide a definition for “on treatment.”

Line 145 A more detailed explanation of how PLWHIV are extracted from the NDB is needed. An operational definition for extraction needs to be presented.

Results

Line 174 How is “regularly visit” defined? There is a need to explain.

Line 180 Table 1 You need to check whether “Total number of regular outpatients” and “Number of outpatients” have changed.

Line 221 In Figure 2, there are 5 lines, but only 4 indicators are attached.

Line 235, Line 239 Are CD4 count<200mm3 and AIDS status based on the time of HIV diagnosis?

Line 227-232 I think it would be a good to go to the Methods section.

Line 241-245 How can the number of patients with CD4 count less than 200 be greater than the number of AIDS? An explanation of how AIDS was defined in this study is needed.

Line 308-310 It is necessary to explain in the Discussion section why there are differences between the NDB and core hospital survey results, and why the relative ratios appear high or low depending on the region.

Minor comments

Line 238 "PLHIV patients" should be modified to "PLHIV".

Line 367 The expression “pandemic” does not seem appropriate for HIV infection.
