# Supplementary material for: Antiretroviral therapies and status of people living with HIV in Japan: An update from hospital survey and national database
Source: PLoS One. 2025 Jan 27;20(1):e0317655. doi: 10.1371/journal.pone.0317655 (PMC11771938; doi:10.1371/journal.pone.0317655)
Supplement: Attachment — Submitted filename: review comment.docx [file pone.0317655.s006.docx]

This study provides valuable data covering a broad range in Japan based on responses from NDB and medical professionals. While the study is generally well-considered, please address the following points:

On line 291, the authors state that "all regions had a treatment introduction rate of ≥94%," which indicates that there are regions where the percentage of patients under treatment falls below 95%. Given that the overall rate exceeds 95%, it can be inferred that regions with rates below 95% likely have fewer patients. Is there a correlation between the small number of patients and the lower proportion of patients under treatment? If possible, please consider adding a discussion that takes into account the regional characteristics.

The vertical axis of Figure 3 shows actual numbers, which makes it difficult to assess the achievement rate of 95-95. If possible, please consider adding a "%" sign to the graph or creating a separate table in the supplementary information.
