# Supplementary material for: Antiretroviral therapies and status of people living with HIV in Japan: An update from hospital survey and national database
Source: PLoS One. 2025 Jan 27;20(1):e0317655. doi: 10.1371/journal.pone.0317655 (PMC11771938; doi:10.1371/journal.pone.0317655)
Supplement: Attachment — Submitted filename: Response Letter.docx [file pone.0317655.s007.docx]

**Response to Reviewers**

First of all, we all thank five reviewers for reviewing our manuscript entitled “Anti-HIV therapies and status of people living with HIV in Japan: An update from hospital survey and National Database.” To facilitate your review of our revisions, the following is a point-by-point response to the questions and comments.

**[Reviewer #1]**

Major comments

- Abstract

AIDS patients -> PLHIV (also in the manuscript)

**Response:** We revised all “AIDS patients” to “PLHIV”.

- Introduction

I think it would be more appropriate to describe the detailed characteristics of NDB in the Methods section rather than in the Introduction section.

**Response:** Thank you for your comment. To focus on the overview and objectives of the NDB, omitting the detailed methods as they were redundant. We moved L66-70 to Method part (Line 140-144) and deleted L71-78.

- Methods

Line 101 Lists of the hospitals that participated in the survey and, if possible, of the respondents are required. It would be good to provide the information as a supplementary table.

**Response:** Given the significant burden of investigating and providing accurate English translations for all the hospitals, we apologize for the inconvenience, but we will include the URL with the list in Japanese in the Fig 1a legend (Line 206-207). Additionally, the location map is available in Fig. 1a of the paper, which we hope you will find helpful.

- Line 127 “Patient retained in care”

Please describe in detail what it means to be linked after diagnosis. For example, is this the same as the definition in the NDB of having visited a hospital or clinic at least once within a 6-month period? Also, I am curious about how cases are handled if PLHIV are transferred to another hospital while receiving treatment at a core hospital.

In addition, please provide a definition for “on treatment.”

**Response:** If diagnosed with HIV, PLHIV will be referred to a medical facility that can provide ART (antiretroviral therapy). The physician at that facility will determine when to start treatment and assist in preparing the necessary documents for applying for a disability certificate, ensuring that financial support is available before the treatment begins. Since the financial support system continues even if the patient transfers to another hospital, PLHIV can continue their treatment with peace of mind.

We provided the definition of “on treatment” in Method part after the definition of “Patient retained in care.” The definition of “Patient on treatment” is HIV-infected person under sustained treatment.

The NDB tracks all medical visits of the same patient, including hospital transfers. Since patients are tracked using a unique ID, one of the strengths of the NDB is its ability to monitor transfers as well.

Line 145 A more detailed explanation of how PLWHIV are extracted from the NDB is needed. An operational definition for extraction needs to be presented.

**Response:** We defined patients who had been prescribed anti-HIV drugs (S1 Table) at least once as PLHIV receiving treatment, and identified all the prescription patterns for these PLHIV. Line 147 has been added.

- Results

Line 174 How is “regularly visit” defined? There is a need to explain.

**Response:** We revised the phrase "regularly visit" to "retained in care".

- Line 180 Table 1 You need to check whether “Total number of regular outpatients” and “Number of outpatients” have changed.

**Response:** Since the Table 1 might be misunderstanding, we revised the table 1.

- Line 221 In Figure 2, there are 5 lines, but only 4 indicators are attached.

**Response:** Thank you for your comment. We revised and added the last indicator "AIDS cases (ACTR)".

- Line 235, Line 239 Are CD4 count<200mm3 and AIDS status based on the time of HIV diagnosis?

**Response:** Yes.

- Line 227-232 I think it would be a good to go to the Methods section.

**Response:** Thank you for your comment. We moved L227-232 to the Methods section (Line 133-138).

- Line 241-245 How can the number of patients with CD4 count less than 200 be greater than the number of AIDS? An explanation of how AIDS was defined in this study is needed.

**Response:** Unlike the CDC's definition of AIDS, in Japan, AIDS is defined as the condition where an individual is infected with HIV and has one or more of the 23 AIDS indicator diseases listed below. Thus, PLHIV with a CD4 count of less than 200 but without AIDS-defining illnesses is not diagnosed with AIDS.

"Indicator Diseases”

A. Fungal Infections

Candidiasis (esophageal, tracheal, bronchial, pulmonary)

Cryptococcosis (excluding lung)

Coccidioidomycosis

(1) Disseminated throughout the body

(2) Occurring in locations other than lung, cervical, or hilar lymph nodes

Histoplasmosis

(1) Disseminated throughout the body

(2) Occurring in locations other than lung, cervical, or hilar lymph nodes

Pneumocystis pneumonia

(Note) The classification name for P. carinii has been changed to P. jirovecii

B. Protozoal Infections

6. Toxoplasmic encephalitis (after 1 month of age)

7. Cryptosporidiosis (with diarrhea lasting over 1 month)

8. Isosporiasis (with diarrhea lasting over 1 month)

C. Bacterial Infections

9. Pyogenic bacterial infections (under 13 years of age, with two or more occurrences of the following within 2 years, due to pyogenic bacteria such as Haemophilus or Streptococcus)

(1) Sepsis

(2) Pneumonia

(3) Meningitis

(4) Osteoarticular infection

(5) Abscess in sites other than the middle ear, skin, mucous membranes, or deep-seated organs

10. Salmonella bacteremia (recurrent, excluding typhoid fever)

11. Active tuberculosis (pulmonary or extrapulmonary) (*)

12. Non-tuberculous mycobacterial infection

(1) Disseminated throughout the body

(2) Occurring in locations other than lung, skin, cervical, or hilar lymph nodes

D. Viral Infections

13. Cytomegalovirus infection (after 1 month of age, affecting organs other than liver, spleen, or lymph nodes)

14. Herpes Simplex Virus Infection

(1) Ulcers of mucous membranes or skin lasting over 1 month

(2) With bronchitis, pneumonia, or esophagitis after 1 month of age

15. Progressive multifocal leukoencephalopathy

E. Tumors

16. Kaposi's sarcoma

17. Primary brain lymphoma

18. Non-Hodgkin lymphoma

19. Invasive cervical cancer (*)

F. Others

20. Recurrent pneumonia

21. Lymphocytic interstitial pneumonia / pulmonary lymphoid hyperplasia: LIP/PLH complex (under 13 years of age)

22. HIV encephalopathy (dementia or subacute encephalitis)

23. HIV wasting syndrome (generalized weakness or wasting syndrome)

- Line 308-310 It is necessary to explain in the Discussion section why there are differences between the NDB and core hospital survey results, and why the relative ratios appear high or low depending on the region.

**Response:** It is believed that the difference arises because, in some regions, the percentage of PLHIV who are visiting core hospitals varies across the area. This contrasts with the results of the NDB, which can track the entire PLHIV population. We added this explanation in the Discussion section (Line 364-367).

Minor comments

- Line 238 "PLHIV patients" should be modified to "PLHIV".

**Response:** We revised all “PLHIV patients” to “PLHIV”.

- Line 367 The expression “pandemic” does not seem appropriate for HIV infection.

**Response:** Thank you for your comment. We revised pandemic to endemic (Line 368).

**[Reviewer #2]**

- The current manuscript lacks sophistication in its writing, leading to important points being obscured. Clear and concise presentation of specific data and results is essential for the reader's understanding.

**Response:** Thank you for your advice. We revised our manuscript with proof reading by native English speaker.

- The objectives and outcome measures of the clinical research are poorly defined. Clear definitions of outcomes such as "treatment success" and "continuous treatment" should be provided, along with detailed results based on these outcomes.

**Response:** Instead of using “treatment success” and “continuous treatment”, we changed those to ”2^nd^ and 3^rd^ of the 90-90-90 target” so that readers can easily understand the objectives of this study.

- The abstract should focus on specific data and results. Abstract and general statements, such as "The NDB is a valuable tool for studying the time course of high-burden diseases such as AIDS and outlining improved health policies." are unnecessary and should be omitted to keep the abstract concise and impactful.

**Response:** We deleted the indicated part.

- The term "core hospitals" appears to be specific to Japan. It is important to clearly explain what constitutes a core hospital, the criteria for their selection, and their role in the healthcare system. This will aid international readers in understanding the context and significance of the data.

**Response:** Core Hospitals were designated by MHLW where ART is provided according to the guidelines. We cited the report previously published about core hospitals by Aikichi Iwamoto et al. (Lines 53-55)

**[Reviewer #3]**

- Page 6, line 122, please add closing parentheses to 4,5, and 6.

**Response:** We added closing parentheses to 4, 5, 6.

- 2. Page 13, line 290, is preefecture prefecture?

**Response:** We corrected the typo.

- 3. Page 14, line 320 to 327, please specify STRs, DVY, TVD, SMT, BVY, and DVT in the main text.

**Response:** We spelled out all the abbreviations in S1 Table. Please refer to the S1 Table.

**[Reviewer #4]**

- Methods: Please mention about the excluding criteria for the collected data.

**Response:** We inserted the following sentence in Method part: Hospitals not informing the number of patients retained after diagnosis, the number of patients on treatment, and the number of patients that were virally suppressed were excluded from our analysis.

- Table1 showed the annual number of all-cause death and it seems that number is increasing in 2020. Do you have any information about age range, nationality, or disease which caused of death for this data?

**Response:** Unfortunately, we do not have additional data supporting the increase death in 2020.

- Figure 1,2,3, are not clearly seen. Please make them more visible.

**Response:** As reviewer#4 mentioned, the figures attached to the manuscript were not clear. However, the figures we submitted through PLOS ONE portal site were clear and had high resolution.

- Figure 2b showed the number of non-Japanese patients were increasing recently. Do you have data of subgroup analysis regarding the rate of sustained treatment and success rate among these population? They may have some difficulties continuing treatment and if there are any difference between Japanese and non-Japanese patients in terms of treatment adherences.

**Response:** Thank you for your valuable feedback. Your question gets to the heart of the matter. Unfortunately, we are unable to obtain information on oral medication adherence from the database. Additionally, we have not conducted any subgroup analyses.

**[Reviewer #5]**

- On line 291, the authors state that "all regions had a treatment introduction rate of ≥94%," which indicates that there are regions where the percentage of patients under treatment falls below 95%. Given that the overall rate exceeds 95%, it can be inferred that regions with rates below 95% likely have fewer patients. Is there a correlation between the small number of patients and the lower proportion of patients under treatment? If possible, please consider adding a discussion that takes into account the regional characteristics.

**Response:** As a reviewer indicated, this is the limitation of our study. Thus, we added the limitation in the Discussion part. (Line 398-399)

- The vertical axis of Figure 3 shows actual numbers, which makes it difficult to assess the achievement rate of 95-95. If possible, please consider adding a "%" sign to the graph or creating a separate table in the supplementary information.

**Response:** To improve visibility, the proportions have been created in the supplemental file. Please refer to S2 Table.
