# Supplementary material for: Antiretroviral therapies and status of people living with HIV in Japan: An update from hospital survey and national database
Source: PLoS One. 2025 Jan 27;20(1):e0317655. doi: 10.1371/journal.pone.0317655 (PMC11771938; doi:10.1371/journal.pone.0317655)
Supplement: Attachment — Submitted filename: Response to Reviewers2.docx [file pone.0317655.s008.docx]

**Response to Reviewers**

**Reviewer #1**

I think the authors have responded faithfully to the comments, and there are no additional comments from me.

**Response:** We would like to thank Reviewer 1 for reviewing our manuscript again and for accepting our revisions.

**Reviewer #2**

Thank you for your revisions; however, after reviewing the updated manuscript, several key areas remain insufficiently addressed, affecting clarity and accessibility for an international audience.

Despite proofreading, the manuscript still lacks the level of clarity and sophistication required for effective communication. Critical points remain unclear, which limits readers’ ability to understand the main conclusions of the study. Additional refinement is necessary to ensure a clear, precise, and engaging presentation of the findings.

**Response:** We would like to thank Reviewer 2 for reviewing our manuscript again and for providing comments, which have considerably helped us improve our manuscript.

As per your comment, we have corrected typos (L50, Table 1), unclear expressions (L252-261), and inconsistencies (L382-386) as identified. We also improved ambiguous terms and syntax (L68, 424-425) and simplified expressions (L243-247).

While you replaced "treatment success" and "continuous treatment" with "90-90-90 target" terminology, this does not clarify the outcome measures. Using this terminology without explicitly defining terms, such as “treatment success” and “continuous treatment,” makes it difficult for readers to interpret the outcomes. Providing precise definitions within the study context would help avoid confusion.

**Response:** The purpose of this study was to report on the second and third targets of the 90-90-90 target, as defined by UNAIDS; therefore, the second and third definitions of the 90-90-90 target are provided (L74-76).

Although the general phrase "the NDB is a valuable tool" was removed, the abstract still lacks conciseness and specificity. It would benefit from focusing exclusively on specific data and results, without general statements, to enhance clarity and impact.

**Response:** Based on your comment, we have removed general wording, subjective evaluations, and redundant phrasing. The abstract has been revised (L23-35).

The term "core hospitals" remains unclear for an international readership. Relying solely on a Japanese-language source to explain the role and criteria of these hospitals limits understanding. It is essential to provide a thorough explanation of the significance and selection criteria of these institutions within the healthcare system. This context would make the manuscript more accessible and meaningful for non-Japanese readers.

**Response:** As the reviewer pointed out, we have created a new section on core hospitals and described how the core hospitals were selected in the Methods section (L84-102).

**Reviewer #3**

The manuscript has been well revised according to the points below.

•Page 6, line 122, please add closing parentheses to 4,5, and 6.

**Response:** We would like to thank Reviewer 3 for reviewing our manuscript again and for providing comments, which have considerably helped us improve our manuscript.

Based on your comment, we have added closing parentheses to 4, 5, 6.

•2. Page 13, line 290, is preefecture prefecture?

**Response:** The typographical error has been corrected now.

•3. Page 14, line 320 to 327, please specify STRs, DVY, TVD, SMT, BVY, and DVT in

the main text.

**Response:** As per your comment, we have spelled out all the abbreviations in S1 Table.

**Reviewer #4**

Dear Dr. Noda

I have thoroughly reviewed the manuscript titled " Anti-HIV therapies and status of people living with HIV in Japan: An update from hospital survey and National Database" submitted to PLOS ONE. After careful consideration, I believe the paper provides valuable insights and contributes to the current understanding of HIV therapies in Japan.

**Response:** We would like to thank Reviewer 4 for reviewing our manuscript again and for accepting our revisions.
